# The effect of caregiver key opinion leaders on increasing caregiver demand for evidence-based practices to treat youth anxiety: protocol for a randomized control trial

**DOI:** 10.1186/s43058-021-00213-x

**Published:** 2021-09-23

**Authors:** Margaret E. Crane, Marc S. Atkins, Sara J. Becker, Jonathan Purtle, Thomas M. Olino, Philip C. Kendall

**Affiliations:** 1 Department of Psychology, Temple University, Weiss Hall, 1701 North 13th Street, Philadelphia, PA 19122 USA; 2 Institute for Juvenile Research, Department of Psychiatry, University of Illinois, Chicago, 1747 West Roosevelt Road, Suite 155, Chicago, IL 60608 USA; 3 Center for Alcohol and Addiction Studies, Brown University School of Public Health, Box G-S121-5, Providence, RI 02912 USA; 4 Department of Health Management and Policy, Dornsife School of Public Health, Drexel University, Nesbitt Hall, Room 351, 3215 Market St, Philadelphia, PA 19104 USA

**Keywords:** Key opinion leader, Theory of planned behavior, Dissemination, Direct-to-consumer marketing, Healthcare utilization, Evidence-based practice, Youth anxiety

## Abstract

**Background:**

Research has identified cognitive behavioral therapy with exposures (CBT) as an effective treatment for youth anxiety. Despite implementation efforts, few anxious youth receive CBT. Direct-to-consumer marketing offers a different approach to address the unmet need for youth receiving effective treatments. Involving a local caregiver key opinion leader in direct-to-consumer initiatives may be an effective strategy to increase caregiver demand for CBT. Research indicates that key opinion leaders improve health promotion campaigns, but key opinion leaders have not been studied in the context of increasing caregiver demand for evidence-based treatments.

**Method:**

Project CHAT (Caregivers Hearing about Anxiety Treatments) will test the role of key opinion leader participation in conducting outreach presentations to increase caregiver desire to seek CBT for their youth’s anxiety. Caregiver attendees (*N* = 180) will be cluster randomized by school to receive one of two different approaches for presentations on CBT for youth anxiety. Both approaches will involve community outreach presentations providing information on recognizing youth anxiety, strategies caregivers can use to decrease youth anxiety, and how to seek CBT for youth anxiety. The researcher-only condition will be co-facilitated by two researchers. In the key opinion leader condition, a caregiver key opinion leader from each local community will be involved in tailoring the content of the presentation to the context of the community, co-facilitating the presentation with a researcher, and endorsing strategies in the presentation that they have found to be helpful. In line with the theory of planned behavior, caregiver attendees will complete measures assessing their knowledge of, attitudes towards, perceived subjective norms about, and intention to seek CBT pre- and post-presentation; they will indicate whether they sought CBT for their youth at 3-month follow-up. Results will be analyzed using a mixed method approach to assess the effectiveness of a key opinion leader to increase caregiver demand for CBT.

**Discussion:**

This study will be the first to examine the potential of key opinion leaders to increase caregiver demand for CBT. If proven effective, the use of key opinion leaders could serve as a scalable dissemination strategy to increase the reach of evidence-based treatments.

**Trial registration:**

This trial was registered on clinicaltrials.gov (NCT04929262) on June 18, 2021. At the time of trial registration, pre/post-presentation data had been collected from 17 participants; thus, it was retrospectively registered.

**Supplementary Information:**

The online version contains supplementary material available at 10.1186/s43058-021-00213-x.

Contributions to the literature

- Direct-to-consumer marketing offers a complimentary approach to provider-focused implementation strategies by increasing consumer demand for evidence-based practices.
- This study will provide data on the effectiveness of key opinion leaders as a strategy for direct-to-consumer dissemination initiatives.
- This study is the first to examine whether direct-to-consumer strategies increase caregiver seeking of cognitive behavioral therapy for youth anxiety.

## Background

Anxiety disorders are highly prevalent among youth, affecting 10–20% of youth [1, 2]. Without intervention, these disorders rarely remit and have adverse sequelae, including educational underachievement, poor social relationships, suicidality, and increased substance use [3–7]. Anxiety disorders create a public health burden from disability, productivity loss, and health care costs [8–10]. Research has identified cognitive behavioral therapy (CBT) as the most effective psychological treatment for youth anxiety [11–13]; exposure is one of the most effective strategies used in CBT for anxiety [11]. Despite research identifying treatments that work, CBT is underutilized in community settings [14]. Only one third of youth with mental health disorders are estimated to receive any treatment [15], and far fewer receive CBT [16]. The majority of dissemination and implementation efforts to increase evidence-based practices (EBPs) use have focused on a “top-down” approach that targets service providers, primarily by increasing the number of practitioners trained in EBPs [14]. Within dissemination and implementation science, dissemination strategies have been understudied relative to implementation strategies [17–19].

### Direct-to-consumer approaches

Direct-to-consumer (DTC) marketing offers a promising approach. DTC dissemination approaches are “bottom-up,” targeting the consumer to improve their understanding of mental health problems, shape their treatment-seeking behavior, and ultimately, increase public demand for CBT [20, 21]. For youth, caregivers are considered the consumer, as they determine and pay for the youth’s services [20, 22, 23]. Although DTC approaches have had success in the pharmaceutical industry [21], this approach is most appropriate for treatments that are safe, supported by research, and underutilized, such as CBT with exposure [24].

### Patient barriers to youth receiving EBPs

DTC initiatives are important given patient barriers to youth receiving treatment. Patient barriers include lack of recognition that treatment is needed, lack of knowledge on how to seek effective treatment, and associated stigma [25–29]. Research suggests that attitudinal barriers (e.g., not perceiving a need for therapy) have a greater impact on treatment utilization than structural barriers (e.g., cost) [30]. Mental health literacy involves the recognition of mental health disorders and knowledge about when and how to seek treatment for them [31]. Caregivers are more likely to seek help if they recognize that their youth has a mental health problem [32, 33]. The belief that therapy will be helpful is associated with the use of mental health services [27, 32, 34], but many individuals do not believe that therapy will be helpful [31]. Of those who want therapy, most do not know how to seek effective treatment [26, 35–37]. Many people hold the belief that treatments are equally effective [24, 31]. Increasing knowledge and awareness of EBPs is key for caregivers to make informed decisions [38, 39].

Increasing knowledge is unlikely to change service-seeking behavior if caregiver stigma about seeking mental health services is not addressed [25, 27, 40]. Caregivers with an anxious child may face family stigma, which arises by association with a stigmatized person [41, 42]. Family stigma can involve public stigma (e.g., stereotypes of blame, shame [41]), and internalized/self-stigma (e.g., self-blame, “bad-caregiver beliefs” [43, 44]). Stigma affects treatment seeking in part because a person may wish to avoid receiving a label of having a mental health issue [45–47]. Some research suggests that increased perceived treatment need and knowledge of how to access treatment at least partially account for the relationship between stigma and treatment seeking [46, 48–50].

### Current direct-to-consumer efforts

DTC programs aim to increase caregivers’ mental health literacy and decrease stigma. Mental health literacy programs for adults have been found to increase knowledge, improve attitudes, and increase help-seeking behaviors [51]. Mental health literacy programs for caregivers have been found to increase caregiver knowledge and self-efficacy about managing their child’s mental health symptoms [52]. Studies have found that stigma is decreased when mental health disorders are portrayed as treatable [46], when knowledge of mental illness is increased [53], and when help-seeking behaviors are enhanced [50, 54]. Peer influence and social norms also play an important role in decreasing stigma [46, 55]. Current DTC efforts to promote EBPs have been limited by a predominant focus on internet educational materials, academic conferences, and outreach presentations [24, 56]. All but one study [57] suggests that brief DTC educational videos increase knowledge, decrease stigma, and increase intention to seek psychological therapy [58–61]. Limitations of previous DTC research include the use of non-representative samples (including undergraduate and Amazon Mturk samples [52, 58–61], and for parent mental health literacy studies, lack of controlled designs [52].

Prior research has shown that DTC marketing effectiveness, mental health stigma, and barriers to treatment vary as a function of demographic factors (education, income, race/ethnicity), youth psychopathology religion, and history of mental health service use [29, 54, 58, 62–68]. Although individual experiences and demographic factors may impact the efficacy of DTC efforts, the majority of DTC efforts involve researchers or therapists spreading knowledge about EBPs without tailoring messaging to local contexts [24, 56].

### Key opinion leaders

Involving a local caregiver key opinion leader (KOL) to tailor DTC initiatives may increase caregiver demand for EBPs. KOLs are trustworthy members of a local community who can use their social influence to enhance the relevance, acceptability, and credibility of DTC initiatives [69–74]. This benefit may be because people are more likely to use interventions that are used by people who are similar to them [75]. KOLs often have a high degree of homophily (i.e., similarity between two individuals) to other members of their social group [76]. Findings from a DTC marketing survey support this approach: results suggest that caregivers would prefer to receive more information about mental health treatments from other caregivers than they currently do [64]. KOLs also could provide personal narratives about strategies used in CBT for youth anxiety, given that narrative stories increase comprehension, interest, and engagement when communicating science to nonexperts [77]. In general, social support and encouragement from others facilitate treatment seeking and decrease stigma [25], and such encouragement is more influential when it comes from a KOL [73]. This may be because KOLs increase the perceived subjective norms about seeking CBT (i.e., belief that other people also value seeking CBT), which would be a factor in predicting caregiver intention to seek CBT according to the theory of planned behavior [78].

KOLs have been found to be an effective strategy in communicating health messages both within healthcare settings and the community [73, 79–81]. Specifically, KOLs increase the likelihood of EBP implementation [73, 80], increase the dissemination of health information [73], and decrease stigma [81, 82]. The involvement of KOLs in DTC efforts harnesses the importance of social relationships in the diffusion of innovations [83]. However, the role of KOLs to increase client demand for mental health EBPs has not been examined.

## Study aims

**Fig. 1:**
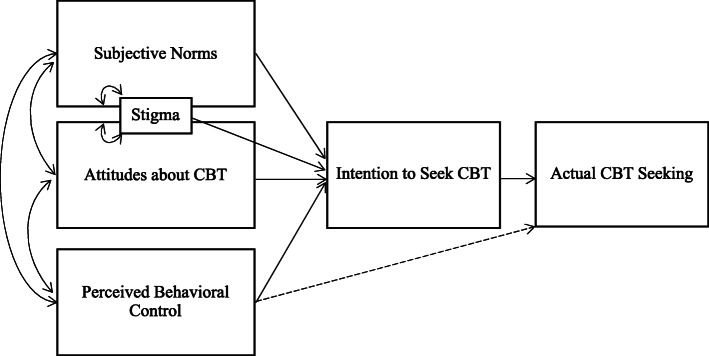
Theory of planned behavior related to seeking cognitive behavioral therapy. *Note*: Figure CC-BY 4.0 Crane, M. E., Atkins, M. A., Becker, S. J., Purtle, J., Olino, T. M., & Kendall, P. C. (2021) doi.org/10.17605/OSF.IO/8X7B4

Project CHAT (Caregivers Hearing about Anxiety Treatments) will evaluate the effects of involving a caregiver KOL in the modification and presentation of an educational outreach presentation for caregivers on youth anxiety by comparing two approaches for outreach presentations about CBT. One presentation will be facilitated by a researcher and a KOL (KOL condition), and the other will be facilitated by two researchers (researcher-only condition). This study uses the theory of planned behavior (see Fig. 1) to evaluate the two presentation conditions. The theory of planned behavior states that attitudes about a behavior, perceived subjective norms about doing a behavior, and perceived behavioral control predict an individual’s intention to complete a behavior, which subsequently predicts their actual behavior [78]. As shown in Fig. 1, stigma (an important barrier to treatment seeking [25, 27, 40]) is conceptualized as being related to both subjective norms and attitudes about CBT. This study will use a mixed methods approach (integrating quantitative and qualitative methods) to test the effect of KOLs on increasing caregiver demand for CBT for youth anxiety through the following aims.

### Aim 1 (Primary aim)

Test the relative effects of researcher-only and KOL conditions on changing caregivers’ intention to seek CBT for their youth, and actual CBT seeking at 3-month follow-up.

*Hypothesis 1a*: Relative to the researcher-only condition, the KOL condition will result in a greater increase in caregiver intention to seek CBT with exposures for their youth.

*Hypothesis 1b*: Relative to the researcher-only condition, the KOL condition will result in a more caregivers seeking of CBT with exposures for their youth at the 3-month follow-up.

### Aim 2 (Secondary aim)

Test the relative effects of researcher-only and KOL conditions on changing caregivers’ (a) perceived subjective norms about seeking CBT, (b) attitudes about CBT, (c) stigma about mental illness, and (d) knowledge of how to seek EBPs.

*Hypothesis 2a*: Relative to the researcher-only condition, the KOL condition will result in a greater (a) increase in subjective norms about seeking CBT, (b) improvement in attitudes about CBT, and (c) decrease in caregiver stigma about mental illness.

*Hypothesis 2b*: Both presentation conditions will result in a similar increase in knowledge about how to seek EBPs.

### Aim 3 (Secondary Aim)

Examine how KOLs affect participants’ impression of the researcher presenter.

*Hypothesis 3*: Participants will view the principal investigator (i.e., the first author) more favorably when she presents with the KOL, relative to when she presents with another researcher.

## Methods/design

### Participants

Participants (*N* = 180) will be primary caregivers who are interested in seeking additional information about youth anxiety; specifically, caregivers who attend a presentation on youth anxiety at their youth’s school. Schools located in a metropolitan area in the northeastern United States will be recruited via their school mental health workers/other school administrators. School administrators will be contacted via email; local school partners (i.e., school psychologists and social workers) will assist with school recruitment as needed. To increase the racial, ethnic, and financial diversity of the sample, schools will only be contacted if they had at least 60% minority student enrollment or at least 60% of students eligible for school lunch [84]. School administrators will advertise presentations as they advertise other school events (e.g., email list, Facebook groups). To be eligible for this study, participants must be least 18 years of age, be fluent in English, be the primary caregiver of a youth aged 5 to 18 years, and have a child at one of the schools offering a presentation. Caregivers will be cluster randomized by school using restricted randomization with Excel’s random number generator. Randomization will occur after the school has agreed to participate in the study, but before caregivers enroll in the study. The principal investigator (clinical psychology candidate with a master’s degree) will randomize schools to the presentation condition and will enroll all participants. Neither the researchers nor the participants are blinded to study condition. Participants will be paid $20 to attend the presentation and complete the pre- and post-presentation questionnaires; $10 to complete the 3-month follow-up questionnaire; and $20 for the qualitative interview.

### Key opinion leaders

To select the KOLs, the principal investigator will contact the school parent-teacher association (or a similar parent group), and ask, “please nominate a caregiver who is well-known and respected within your community, and who reflects the diversity of the school as a whole.” If a school does not have an active parent-teacher association (or similar group of active parents), the school staff may select the KOL. The KOLs do not necessarily have to be a member of the parent-teacher association or have experience (professional or personal) with mental health. Previous research supports KOL nomination by knowledgeable community members (e.g., caregivers in the parent-teacher association) as a valid method for identifying trusted individuals in the community [74, 85]. The principal investigator will ask if the first KOL on the list is interested in participating in the project. The KOL must be willing to endorse CBT with exposures. Should the KOL decline to participate, the parent-teacher association will be asked to nominate a second caregiver KOL. There will be one KOL per school; the total number of KOLs will depend on the number of schools needed to recruit 180 caregiver participants.

KOLs from at least two schools in the KOL condition will participate in a 2-h feedback meeting with the principal investigator, with the goal of leveraging the KOLs to be champions of CBT. The KOLs will be sent a draft of the presentation to review prior to the meeting. During this meeting, the KOLs will discuss their experiences with youth anxiety, factors about their communities that may affect how anxiety symptoms present or are understood, and how caregivers in their community typically seek therapy. The principal investigator will review the presentation materials and encourage the KOLs to discuss their reactions and provide feedback. The KOLs will consider which strategies they can endorse as being effective (e.g., remaining calm when their child becomes emotional). Motivational interviewing techniques will be used should KOLs be skeptical about the value of CBT [86]. The principal investigator will then modify the outreach presentation based on KOL feedback. Presentations will be modified separately for each school, so the KOLs who meet together do not need to come to consensus on presentation content. Following the group KOL feedback meeting, the principal investigator will meet with each KOL individually to review/approve the modifications made; answer remaining KOL questions about the content; determine which sections the KOL is comfortable presenting, and which strategies they are willing to endorse; and give the KOL the opportunity to practice. KOL meetings will take place via zoom. KOLs will be paid $40 per hour (5 h = $200 per KOL).

The KOL training checklist will be used to ensure that the KOL training is delivered consistently (see Additional file 1). The principal investigator will complete this checklist following the KOL training. She will mark whether the group training discussed KOL experiences with youth anxiety and reviewed the presentation materials, as well as whether the phone call reviewed modifications made to the presentation, allowed the KOL to ask questions, determined which parts of the presentation the KOL will present and which strategies the KOL will endorse, and allowed the KOL the chance to practice.

### Outreach conditions

Caregivers in both conditions will be invited to an outreach presentation, which lasts 75 min with an additional 15 min for caregiver questions. Presentations will occur in the evening via Zoom, separate from parent-teacher association meetings. Each presentation will include information about identifying anxiety disorders, strategies for caregivers to help their youth with anxiety, CBT for youth anxiety, and strategies for finding a therapist who uses CBT with exposures. Exposure therapy will be emphasized given that exposure therapy is underutilized by therapists in the community despite being a core ingredient of CBT [87]. The text on the presentations is written at a 5.3 grade reading level. Presentations will incorporate stigma reduction strategies, such as education to dispel myths, and behavioral decision-making tools to elicit hope, empowerment, and motivation [41, 88, 89]. Presentation content is manualized and is presented using PowerPoint.

#### Researcher-only condition

Half the schools will be cluster randomized to receive a researcher-facilitated presentation, led by two clinical psychology graduate students (the principal investigator and another graduate student). Content will be the same for all schools randomized to the researcher-only condition. This is an active control condition. Researcher-facilitated outreach presentations are a current strategy research groups use to disseminate information to the community [24, 56, 90].

#### KOL condition

The other half of the schools will receive KOL co-facilitated presentations with the principal investigator (a clinical psychology PhD candidate). The KOLs will be introduced as a member from their school who has worked with the principal investigator to tailor the presentation to their community. Although the presentation is manualized and will contain the same core principles, content may vary by school in terms of specific examples and content emphasized based on KOL feedback. KOLs will be encouraged to share personal stories and examples of how the presentation material can apply to the school community to increase a sense of homophily to the KOL, as well as local relevance of the information.

#### Fidelity and manipulation checks

A 20-item Knowledge Test will assess caregivers’ knowledge of the content reviewed in the presentation (i.e., identifying anxiety disorders, strategies for caregivers to manage youth anxiety, EBPs to treat youth anxiety, and strategies for finding a therapist). The knowledge test is modeled after one to assess therapist training of CBT for anxiety [91]. Questions are true/false and multiple-choice format. Responses will be coded such that 1 = correct and 0 = incorrect, for a maximum of 20 points. The Knowledge Test will be used as a manipulation check to test participants’ understanding of the presentation material.

A content checklist will assess the core components of the presentation (i.e., identifying anxiety disorders, strategies for caregivers to help their youth with anxiety, how anxiety is treated, and strategies for finding a CBT therapist). A research assistant will function as an independent evaluator to complete this measure and evaluate the content of the outreach presentations. The research assistant will code for presenter and audience member self-disclosure about experience receiving therapy for themselves or their child. The research assistant also will record the total amount of time each presenter speaks. Two research assistants will be present for at least 20% of presentations; interrater reliability of the evaluators (*κ*) will be calculated.

### Quantitative measures

**Fig. 2:**
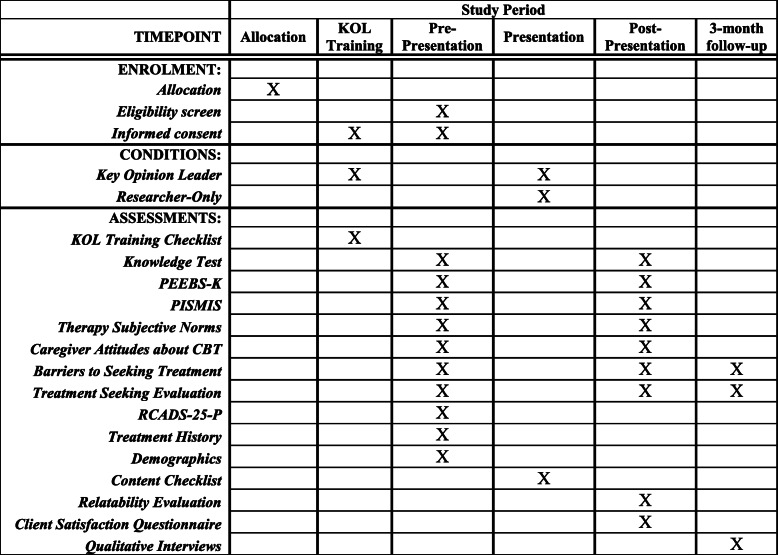
SPIRIT: Schedule of Enrolment, Interventions, and Assessment

All questionnaires will be completed and stored on REDCap (a HIPAA secure platform [92]) hosted at Temple University. Participants will provide informed consent via REDCap before completing questionnaires. Figure 2 provides a summary of the schedule of enrolment, interventions, and assessments using the SPIRIT flow diagram [93]. All measures that were created for Project CHAT are in Additional file 1 and are described below.

#### Treatment seeking evaluation

Pre- and post-presentation, caregivers will rate how likely they are to both seek a therapist for their child, as well as a therapist who uses exposure therapy, in the next 3 months. Rating scale ranges from 1 (*very unlikely*) to 5 (*very likely*). At the 3-month follow-up assessment, parents will be asked if they have sought therapy for their youth since the presentation. If so, they will be asked if the child has started therapy, if they requested a therapist who uses exposure therapy, and for the name of their child’s therapist.

#### Knowledge about seeking CBT

The Parent Engagement in Evidence-Based Services Questionnaire [94] is a 39-item measure of factors associated with seeking mental health care based on the theory of planned behavior [78]. Caregivers rate each statement on a 5-point Likert scale ranging from 1 (*strongly disagree*) to 5 (*strongly agree*); some items are reverse coded. Caregiver ratings are summed to create five subscales [95]; this study will use the knowledge subscale to measure caregivers’ perceived understanding of how to seek EBPs (i.e., perceived behavioral control). On this subscale, higher scores indicate higher levels of perceived knowledge about seeking evidence-based practice. Evidence supports knowledge subscale’s internal consistency (*α* = .72) and convergent validity (*r* = .25–.41) [95].

#### Internalized stigma

The Parents’ Internalized Stigma of Mental Illness Scale [96] is a 10-item measure of caregiver perception of internalized stigma for having a youth with a mental illness. Caregivers rate each statement on a 4-point Likert scale ranging from 1 (*strongly disagree*) to 4 (*strongly agree*); some items are reverse coded. Higher scores indicate higher levels of family stigma. The Parents’ Internalized Stigma of Mental Illness Scale has acceptable internal consistency (*α* = .76). It is an adaptation of the well-validated Internalized Stigma of Mental Illness Scale [97, 98], which has demonstrated sensitivity to change in the expected direction after stigma reduction interventions [97].

#### Caregiver attitudes about cognitive behavioral therapy

The Caregiver Attitudes about CBT includes 18 strategies used in CBT for youth anxiety. Caregivers rate how helpful they believe each strategy would be for treating their child on a five-point scale ranging from 1 (*very unhelpful*) to 5 (*very helpful*). All items will be summed; higher scores indicate more favorable attitudes. Items were generated using an expert consensus (three clinical psychologists specializing in exposure treatment and one advanced doctoral candidate in clinical psychology). Some items were modeled on the Knowledge of Evidence-Based Services Questionnaire [99].

#### Therapy subjective norms

The Therapy Subjective Norms Questionnaire is a six-item measure of caregiver perception of subjective norms for seeking therapy. It was modeled from previously used measures of subjective norms [100, 101]. Caregivers rate each item on seven-point scale ranging from 1 (*strongly disagree*) to 7 (*strongly agree*). Three items assess injunctive norms (i.e., how other people would view an action the participant does; injunctive norms subscale), and three items assess descriptive norms (i.e., the participant’s view about what other people are doing; descriptive norms subscale). Items will be summed to create a score for overall subjective norms (all six items), as well as the injunctive and descriptive norms subscales; higher scores indicate more positive subjective norms about seeking therapy. Participants will complete two versions of this measure (12 items total): in one version, they will rate subjective norms related to seeking therapy, and in the other version, they will rate subjective norms related to seeking CBT.

#### Impression of presenters

On the Relatability Evaluation, caregivers will rate each presenter (the KOL and the researcher, or the two researchers) on the following 10 dimensions: relatability, likeability, similarity, similarity in thinking, similarity of beliefs, credibility, trustworthiness, understanding of the local community, familiarity, and friendship. Scores will be given on a scale ranging from 1 (*strongly disagree*) to 5 (*strongly agree*). These items are based on characteristics of homophily [102].

#### Barriers to seeking treatment

The Barriers to Seeking Treatment questionnaire asks participants to indicate whether they agree with 21 potential barriers to treatment (yes/no). This questionnaire is adapted from the Collaborative Psychiatric Epidemiology Studies [103] and includes items relating to attitudinal barriers (e.g., wanted to handle on their own, stigma) and structural barriers (e.g., cost, transportation) [29]. Items were adapted to describe potential barriers caregivers may face (e.g., rather than saying “I need therapy,” the questionnaire was modified to read “my child needs therapy”).

#### Youth anxiety

The Brief Revised Child Anxiety and Depression Scale-Parent Version is a 25-item caregiver report measure of anxiety and depressive symptoms [104]. Items are rated on a 4-point Likert scale from 0 (*never*) to 3 (*always*). It yields three scores: Total Anxiety, Total Depression, and Total Anxiety and Depression. This study will use the Total Anxiety score. Previous research supports the Brief Revised Child Anxiety and Depression Scale total anxiety subscale’s internal reliability (*α* = .80–.86), retest reliability (*r* = .85), convergent validity (*r* = .59), and discriminant validity for anxiety diagnoses (AUC = .81) [104].

#### Client satisfaction

Caregivers will evaluate their satisfaction with the presentation using the Client Satisfaction Questionnaire [105]. This scale includes eight Likert scale questions and three short answer questions. On the Likert scale questions, caregivers will rate their level of satisfaction on a 4-point scale ranging from 1 to 4, with higher composite scores indicating greater program satisfaction. Psychometric analyses indicate excellent internal consistency (*α* = .93) and convergent validity (*r* = − .40–.23) [105].

#### Demographics and mental health history

A demographics questionnaire will assess caregiver and youth age, gender, race, ethnicity, and country of origin; caregiver level of education, income, and religious service attendance; and youth health insurance status. The presenters also will indicate their age, gender, country of origin, number of children, and level of education to assess their similarity to participants. On the mental health history questionnaire, participants will indicate whether they or their youth have ever been diagnosed with or treated for a mental disorder, whether they or their youth have received CBT with exposures, and their level of satisfaction with their youth’s previous treatment experience.

### Qualitative interviews

After participants have completed the 3-month follow-up questionnaire, 40 participants will be contacted to complete a qualitative interview via a Zoom videoconference. Participants will be purposefully sampled such that 20 participants who have sought treatment (10 per condition) and 20 who have not sought treatment (10 per condition) will be selected using Excel’s random number generator. Additional participants will be recruited until thematic saturation is reached [106].

Semi-structured interviews (see Additional file 1 for the interview guide) will be conducted by undergraduate research assistants (*N* = 3) using a funnel approach, with open ended questions followed by specific required and optional probes for details [107]. Interviews will elicit information about barriers to seeking treatment, and the role of the presentation in reducing those barriers. Primary topics will include: (1) their perception of the presenters; (2) ways in which the presenters affected their decision to seek treatment; (3) factors they considered when seeking treatment; (4) strategies they have used from the presentation; (5) their perception of exposure therapy; and (6) general ways that the mental health system could be improved to improve access to therapy. Interviews will close with a question asking for general additional feedback. Interviews will last approximately 30 min and will be digitally recorded via Zoom.

After each interview, the interviewer will rate the participant’s level of interest and involvement in answering the questions (1 = *very low* to 5 = *very high*), their understanding of the interview (1 = *limited* to 5 = *complete*), and their impression of the participant’s knowledge of the topics discussed (1 = *highly questionable* to 5 = *highly knowledgeable*). The interviewer also will comment on discrepancies in the interview and circumstances that may have affected quality of responses. Zoom transcripts of the interviews will be used, and a research assistant will check the transcription for accuracy. Transcripts will be deidentified.

### Analytic plan

#### Missing data

The primary analytic tool will be multilevel modeling using maximum likelihood estimation, which provides unbiased parameter estimates when data are missing at random. The missing at random assumption will be tested by multiple logistic regression analyses examining whether key predictors at baseline (i.e., Knowledge Test, Parent Engagement in Evidence-Based Services Questionnaire–Knowledge subscale, Parents’ Internalized Stigma of Mental Illness Scale, Therapy Subjective Norms, Caregiver Attitudes about CBT, Treatment Seeking Evaluation, and demographics) are associated with study retention. Should analyses reveal that dropout is differentially associated with outcomes, multiple imputation will be used [108–110]. Every effort will be made to prevent missing data, such as by using REDCap options that remind participants to answer blank questions, and by emailing participants who have not completed all questionnaires.

#### Power analysis

For Primary Aim 1, a Monte Carlo-based power estimate was derived using Mplus with 10,000 replications. For the sample size of 180, assuming a Type I error rate of 5%, a two-tailed test, statistical power was .83 to detect a medium-sized effect (*r* = .30) of randomization group on longitudinal changes, given an expectation of a small (*r* = .15) effect for the control group. For Primary Aim 2, power was calculated using G*Power. Given the brevity of the 3-month follow-up questionnaire, a 10% attrition rate was assumed. Assuming a Type I error rate of 5%, a two-tailed test, and a 25% rate of seeking CBT in the researcher-only condition, statistical power was .82 to detect a medium effect (odd ratio = 1.72).

#### Data analysis and interpretation

##### Quantitative analyses

Quantitative analyses will use multilevel modeling to account for the nesting of repeated measures within caregivers. Preliminary analyses will examine the effect of clustering of caregivers within schools. If schools account for more than 10% of variance in the outcomes after controlling for condition, a three-level multilevel model will be used to account for nesting of repeated measures within caregivers within schools.

Analyses will consider intention to seek CBT with exposures (Treatment Seeking Evaluation - Intention to seek CBT), subjective norms about seeking CBT (Therapy Subjective Norms Questionnaire–CBT), attitudes about CBT (Caregiver Attitudes about CBT), caregiver stigma about mental illness (Parents’ Internalized Stigma of Mental Illness Scale), and knowledge about how to seek EBPs for youth anxiety (Parent Engagement in Evidence-Based Services Questionnaire–Knowledge Subscale) as person-level dependent factors; condition (caregiver or researcher co-facilitator) as a person-level predictor; and time (pre- and post-presentation) as an observation-level predictor. In separate multilevel models, (a) intention to seek CBT, (b) Therapy Subjective Norms Questionnaire–CBT, (c) Caregiver Attitudes about CBT, (d) Parents’ Internalized Stigma of Mental Illness Scale, and (e) Parent Engagement in Evidence-Based Services Questionnaire–Knowledge subscale will be regressed on time, condition, and the interaction between time and condition; a random intercept will be included in all five multilevel models. A binary logistic regression will be conducted with CBT service seeking at the 3-month follow-up (Treatment Seeking Evaluation - Actual CBT seeking) entered as the dependent variable, condition entered as the independent variable, and youth anxiety (Brief Revised Child Anxiety and Depression Scale–Total Anxiety) entered as a control variable. *T*-tests will be used to compare difference between conditions for each item on the Relatability Evaluation of the principal investigator. This study will examine caregiver demographic factors, youth anxiety (Brief Revised Child Anxiety and Depression Scale–Total Anxiety), racial similarity to the presenter (Demographics, same race), and self-disclosure (Content Checklist, self-disclosure) as potential moderators of the effect of presentation condition on intention to seek CBT. In separate multilevel models, intention to seek CBT will be regressed on time, condition, each potential moderators, and their three-way interaction.

##### Qualitative analyses

The transcribed qualitative interviews will be entered into NVivo software for analysis. Qualitative analyses will use a direct content analysis approach [111]. The coding team will create an initial codebook using the primary topics asked in the qualitative interviews. Additional codes will be added to code text that does not fit into the initial categories, to split the initial codes into two, or to create new codes. Coding will occur through a consensus process in which each transcript will be coded independently by two raters, who will arrive at consensus through discussion as needed [112]. Thematic responses will be examined by both condition and by whether the caregiver has sought treatment for their youth (4 groups total).

##### Integration procedures

Mixed methods integration will follow a QUAN ➔ qual structure with an expansion approach [113]; quantitative methods are used to test hypotheses about the intervention and qualitative methods are used to contextual the results.

## Trial status

The Institutional Review Board at Temple University has approved all study procedures. Recruitment and data collection for this study began in May 2021. At the time of publication, 17 participants attended the presentation and completed the post-presentation assessment; 4 participants have completed the 3-month follow-up data collection.

## Considerations and limitations

Several considerations were made in selecting the study methods. First, we considered recruiting KOLs with lived experience as a parent of a child with anxiety; contact strategies with individuals with lived experience are a common strategy to destigmatize mental illness [114]. However, we determined that individuals with lived experience and KOLs may be two different groups of people, and thus we decided not to require lived experience among KOLs. Future research could compare KOLs versus individuals with lived experience for increasing the caregiver demand of EBPs. Second, we considered whether school staff could select KOLs. However, we decided that fellow caregivers would likely have a better sense of which caregivers were respected by their peers, rather than the caregivers known to school staff. The limitation of this approach is that some schools may not have active parent-teacher association (or similar parent groups), thus limiting the feasibility of a consistent KOL selection process. Third, we set the follow-up period as 3 months to give individuals enough time to seek treatment, but not too much time such that they would forget aspects of the presentation that will be discussed in the qualitative interviews. A limitation of this approach is that it is possible that caregivers may take more than 3 months to seek treatment for their child, which will not be captured by this research study. Fourth, the impact of COVID-19 on both schools and families may negatively affect recruitment of schools and caregivers. To mitigate this challenge, the research team has established partnerships with school mental health workers who are assisting with school recruitment.

## Discussion

This project will evaluate a DTC strategy to increase caregiver seeking of CBT for youth anxiety. Most dissemination and implementation efforts examine strategies to increase the use of CBT by providers [14]. However, these efforts do not necessarily affect initial treatment seeking [24]. Educational outreach strategies are used to increase demand for EBPs [24, 56, 90], but their efficacy has yet to be evaluated. Further, although exciting work has begun to examine DTC efforts for increasing demand of EBPs [58–61, 65], no study has examined the effect of DTC efforts on actual treatment seeking behavior. Additionally, although research indicates that KOLs improve health promotion campaigns [73], their efficacy in increasing uptake of EBPs in clinical psychology has not been examined. Previous research on patient and public involvement in research has largely been qualitative, and randomized trials have focused only on one type of engagement outcome, such as participant recruitment [115]. This project also will examine how the presence of a local stakeholder (i.e., a KOL) affects participants’ perceptions of a researcher. Together, the findings from the study will inform future dissemination initiatives to increase client demand for EBPs.

## Supplementary Information

**Additional file 1.**

## Data Availability

Not applicable

## Abbreviations

CBT: Cognitive behavioral therapy
DTC: Direct-to-consumer
EBP: Evidence-based practice
KOL: Key opinion leader
Project CHAT: Caregivers Hearing about Anxiety Treatments
