# Supplementary material for: The effect of caregiver key opinion leaders on increasing caregiver demand for evidence-based practices to treat youth anxiety: protocol for a randomized control trial
Source: Implement Sci Commun. 2021 Sep 23;2:107. doi: 10.1186/s43058-021-00213-x (PMC8460198; doi:10.1186/s43058-021-00213-x)
Supplement: Supplementary file 1 — Additional file 1. [file 43058_2021_213_MOESM1_ESM.pdf]

**Additional File 1**

Measures Created for Project CHAT

Crane, M. E., Atkins, M. A., Becker, S. J., Purtle, J., Olino, T. M., & Kendall, P. C. (2021). The effect of caregiver key opinion leaders on increasing caregiver demand for evidence-based practices to treat youth anxiety: Protocol for a randomized control trial. *Implementation Science Communications.*

## Treatment Seeking Evaluation

**<u>Pre- and post-presentation</u>**
For the following questions, please consider your child (initials:____)

In the next three months, how likely are you to seek a therapist for your child?
(1) Very unlikely
(2) Unlikely
(3) Neither likely nor unlikely
(4) Likely
(5) Very likely

In the next three months, how likely are you to seek a therapist who uses <u>exposure therapy</u> (i.e., slowly helps a child face their fears) for your child?
(1) Very unlikely
(2) Unlikely
(3) Neither likely nor unlikely
(4) Likely
(5) Very likely

**<u>3-month follow-up</u>**
*For the following questions, please consider your child with initials, [child initials].*

Have you sought therapy for your child?
- ☐ Yes
- ☐ No

*<u>Yes, sought therapy for child</u>*

Has your child started therapy?
- ☐ Yes, they have had their first appointment
- ☐ They haven't started, but their first appointment is scheduled
- ☐ No, they don't have an appointment scheduled

When you were looking for a therapist, did you request a therapist who uses <u>exposure therapy</u> (i.e., slowly helps a child face their fears)?
- ☐ Yes
- ☐ No
- ☐ Unsure

*We are interested in learning more about the therapeutic approach your therapist uses.*
What is the name of your child's therapist? ______

What clinic does your child's therapist work at? ______

CC-BY 4.0 Crane, M. E., Atkins, M. A., Becker, S. J., Purtle, J., Olino, T. M., & Kendall, P. C. (2021)
doi.org/10.17605/OSF.IO/8X7B4

## Caregiver Attitudes about Cognitive Behavioral Therapy

The items below describe strategies that may be used in therapy for youth with anxiety. Please rate how **helpful** you think each strategy would be if you were seeking treatment for your child's anxiety.

| 1 | 2 | 3 | 4 | 5 |
|---|---|---|---|---|
| Very unhelpful | Unhelpful | Neutral | Helpful | Very helpful |

| | | | | | | |
|---|---|---|---|---|---|---|
| 1. | Therapist provides information about normal anxiety level in children. | 1 | 2 | 3 | 4 | 5 |
| 2. | Therapist helps your family identify how your child's anxiety is getting in the way for your child/for your family | 1 | 2 | 3 | 4 | 5 |
| 3. | Therapist teaches you and your child about the difficulties the child is having, and how therapy activities are meant to help. | 1 | 2 | 3 | 4 | 5 |
| 4. | Therapist creates an individualized treatment plan for your child. | 1 | 2 | 3 | 4 | 5 |
| 5. | Therapist sets specific therapy goals in collaboration with you and your child. | 1 | 2 | 3 | 4 | 5 |
| 6. | Therapist asks you and your child to fill out questionnaires weekly to monitor how your child is doing. | 1 | 2 | 3 | 4 | 5 |
| 7. | Therapist coaches you to use therapy skills at home. | 1 | 2 | 3 | 4 | 5 |
| 8. | Therapist demonstrates a behavior they want your child to do. | 1 | 2 | 3 | 4 | 5 |
| 9. | Therapist gives your child activities to practice therapy skills between sessions. | 1 | 2 | 3 | 4 | 5 |
| 10. | Therapist teaches the child to identify and effectively communicate their feelings. | 1 | 2 | 3 | 4 | 5 |
| 11. | Therapist teaches your child to relax their body. | 1 | 2 | 3 | 4 | 5 |
| 12. | Therapist teaches the child to identify anxious thoughts, consider how true an anxious thought is, and come up with a more realistic or helpful thought. | 1 | 2 | 3 | 4 | 5 |
| 13. | Therapist teaches your child problem solving. | 1 | 2 | 3 | 4 | 5 |
| 14. | Therapist rewards your child for brave/desirable behavior. | 1 | 2 | 3 | 4 | 5 |
| 15. | Therapist teaches you to reward your child for brave/desirable behavior. | 1 | 2 | 3 | 4 | 5 |
| 16. | Therapist teaches you to pay more attention to positive behavior and to ignore minor distress or misbehavior. | 1 | 2 | 3 | 4 | 5 |
| 17. | Therapist supports your child to help them slowly face their fears. | 1 | 2 | 3 | 4 | 5 |
| 18. | Therapist helps your child plan how to address future problems to prevent difficulties from coming back. | 1 | 2 | 3 | 4 | 5 |

CC-BY 4.0 Crane, M. E., Frank, H. E., & Kendall, P. C. (2021) doi.org/10.17605/OSF.IO/8X7B4

**Scoring**

- Sum all items

CC-BY 4.0 Crane, M. E., Frank, H. E., & Kendall, P. C. (2021) doi.org/10.17605/OSF.IO/8X7B4

## Therapy Subjective Norms

**Therapy**

Please rate your level of agreement on each statement using this scale:

| 1 | 2 | 3 | 4 | 5 | 6 | 7 |
|---|---|---|---|---|---|---|
| Strongly disagree | | | Neutral | | | Strongly agree |

Injunctive norms

If my child needed help for anxiety, most people who are important to me would…

| | | | | | | | | |
|---|---|---|---|---|---|---|---|---|
| 1. Think I should take my child to a therapist. | 1 | 2 | 3 | 4 | 5 | 6 | 7 |
| 2. Approve of me taking my child to a therapist. | 1 | 2 | 3 | 4 | 5 | 6 | 7 |
| 3. Want me to take my child to a therapist. | 1 | 2 | 3 | 4 | 5 | 6 | 7 |

Descriptive norms

If another caregiver's child had anxiety…

| | | | | | | | |
|---|---|---|---|---|---|---|---|
| 4. Most caregivers who are important to me would take their child to a therapist. | 1 | 2 | 3 | 4 | 5 | 6 | 7 |
| 5. Most caregivers who are like me would take their child to a therapist. | 1 | 2 | 3 | 4 | 5 | 6 | 7 |
| 6. Other caregivers who are like me have taken their child to a therapist. | 1 | 2 | 3 | 4 | 5 | 6 | 7 |

**Cognitive Behavioral Therapy**

Cognitive-behavioral therapy has been shown to be the most effective therapy for youth anxiety. In this therapy, youth learn to coping skills and practice using their coping skills by slowly facing their fears.

Injunctive norms

If my child needed help for anxiety, most people who are important to me would…

| | | | | | | | |
|---|---|---|---|---|---|---|---|
| 1. Think I should take my child to a therapist who uses cognitive behavior therapy. | 1 | 2 | 3 | 4 | 5 | 6 | 7 |
| 2. Approve of me taking my child to a therapist who uses cognitive behavior therapy. | 1 | 2 | 3 | 4 | 5 | 6 | 7 |
| 3. Want me to take my child to a therapist who uses cognitive behavior therapy. | 1 | 2 | 3 | 4 | 5 | 6 | 7 |

Descriptive norms

If another caregiver's child had anxiety…

| | | | | | | | |
|---|---|---|---|---|---|---|---|
| 4. Most caregivers who are important to me would take their child to a therapist who uses cognitive behavior therapy. | 1 | 2 | 3 | 4 | 5 | 6 | 7 |
| 5. Most caregivers who are like me would take their child to a therapist who uses cognitive behavior therapy. | 1 | 2 | 3 | 4 | 5 | 6 | 7 |
| 6. Other caregivers who are like me have taken their child to a therapist who uses cognitive behavior therapy. | 1 | 2 | 3 | 4 | 5 | 6 | 7 |

CC-BY 4.0 Crane, M. E., Atkins, M. A., Becker, S. J., Purtle, J., Olino, T. M., & Kendall, P. C. (2021)
doi.org/10.17605/OSF.IO/8X7B4

**Scoring**

- Items are summed to create a score for overall subjective norms (all six items), as well as the injunctive and descriptive norms subscales (three items each)

CC-BY 4.0 Crane, M. E., Atkins, M. A., Becker, S. J., Purtle, J., Olino, T. M., & Kendall, P. C. (2021)
doi.org/10.17605/OSF.IO/8X7B4

**Relatability Evaluation**

We would love to hear more about your thoughts on ___________________________.
Please use the following scale to rate the statements below.

| 1 | 2 | 3 | 4 | 5 |
|---|---|---|---|---|
| Strongly disagree | Disagree | Neutral | Agree | Strongly Agree |

| | | | | | | | |
|---|---|---|---|---|---|---|---|
| 1. | I could relate to the presenter. | 1 | 2 | 3 | 4 | 5 |
| 2. | The presenter was likeable. | 1 | 2 | 3 | 4 | 5 |
| 3. | The presenter was similar to me. | 1 | 2 | 3 | 4 | 5 |
| 4. | The presenter thinks like me. | 1 | 2 | 3 | 4 | 5 |
| 5. | The presenter and I have similar beliefs about mental health. | 1 | 2 | 3 | 4 | 5 |
| 6. | The presenter was a credible source of information about mental health. | 1 | 2 | 3 | 4 | 5 |
| 7. | The presenter was trustworthy. | 1 | 2 | 3 | 4 | 5 |
| 8. | The presenter understood my community. | 1 | 2 | 3 | 4 | 5 |
| 9. | Before the presentation, I was familiar with the presenter. | 1 | 2 | 3 | 4 | 5 |
| 10. | Before the presentation, I was friends with the presenter. | 1 | 2 | 3 | 4 | 5 |

CC-BY 4.0 Crane, M. E., Atkins, M. A., Becker, S. J., Purtle, J., Olino, T. M., & Kendall, P. C. (2021)
doi.org/10.17605/OSF.IO/8X7B4

**Mental Health History**

<u>Caregiver mental health history:</u>
*Please answer the following questions based on YOUR history.*

Have you ever been diagnosed with a mental disorder?
- o Yes
- o No

Have you seen a mental health professional?
- o Yes
- o No

*If yes*:
Did your therapist do cognitive behavioral therapy?
- o Yes
- o No
- o Unsure

Did your therapist use exposures (i.e., slowly helped you face your fears)?
- o Yes
- o No
- o Unsure

Please rate your overall level of satisfaction with your previous therapy experience(s):
- (1) Very dissatisfied
- (2) Dissatisfied
- (3) Neither satisfied nor dissatisfied
- (4) Satisfied
- (5) Very satisfied

<u>Child mental health history:</u>
*Please answer the following questions based on your child's (initials:_____) history.*

Has your child ever been diagnosed with a mental disorder?
- ☐ Yes
- ☐ No

CC-BY 4.0 Crane, M. E., Atkins, M. A., Becker, S. J., Purtle, J., Olino, T. M., & Kendall, P. C. (2021)
doi.org/10.17605/OSF.IO/8X7B4

Has your child seen a mental health professional?

- ☐ Yes
- ☐ No

*If yes*:
Did their therapist do cognitive behavioral therapy?

- o Yes
- o No
- o Unsure

Did their therapist use exposures (i.e., slowly helped your child face their fears)?

- o Yes
- o No
- o Unsure

Please rate your overall level of satisfaction with your child's previous therapy experience(s):

(1) Very dissatisfied
(2) Dissatisfied
(3) Neither satisfied nor dissatisfied
(4) Satisfied
(5) Very satisfied

CC-BY 4.0 Crane, M. E., Atkins, M. A., Becker, S. J., Purtle, J., Olino, T. M., & Kendall, P. C. (2021)
doi.org/10.17605/OSF.IO/8X7B4

## Demographic Questionnaire

<u>Caregiver demographics</u>

**For the following questions, please enter/select your demographic information. As a reminder, all information collected remains confidential.**

Age (in years): ___________

What sex were you assigned at birth?

- ☐ Male
- ☐ Female
- ☐ A sex not listed:_____
- ☐ Prefer not to say

How would you describe your gender? (check all that apply)

- ☐ Agender
- ☐ Cisgender
- ☐ Female
- ☐ Genderqueer
- ☐ Male
- ☐ Non-binary
- ☐ Transgender
- ☐ A gender not listed:______
- ☐ Prefer not to say

Sexual orientation (check all that apply):

- ☐ Asexual
- ☐ Bisexual
- ☐ Gay or Lesbian
- ☐ Queer
- ☐ Straight/heterosexual
- ☐ A sexual orientation not listed:______
- ☐ Prefer not to say

CC-BY 4.0 Crane, M. E., Atkins, M. A., Becker, S. J., Purtle, J., Olino, T. M., & Kendall, P. C. (2021)
doi.org/10.17605/OSF.IO/8X7B4

Race (check all that apply):

- ☐ American Indian or Alaska Native
- ☐ Asian
- ☐ Black or African American
- ☐ Native Hawaiian or Other Pacific Islander
- ☐ White
- ☐ A race not listed:______
- ☐ Prefer not to say

Are you of Hispanic, Latino/a/x, or of Spanish origin? (check all that apply)

- ☐ No, not of Hispanic, Latino/a/x, or Spanish origin
- ☐ Yes, Cuban
- ☐ Yes, Mexican, Mexican American, Chicano/a/x
- ☐ Yes, Puerto Rican
- ☐ Yes, another Hispanic, Latino/a/x or Spanish origin
- ☐ An ethnicity not listed:_____
- ☐ Prefer not to say

Where were you born?

- o In the United States
- o In another country
- o Prefer not to say

Relationship status (check all that apply):

- ☐ Divorced
- ☐ Married, currently living separate from partner
- ☐ Married, currently living with partner
- ☐ Not married, currently living with partner
- ☐ Remarried
- ☐ Single, never married
- ☐ Widow/widower
- ☐ Relationship status not listed:______
- ☐ Prefer not to say

Which of the following best describes your relationship to your child?

- o Adoptive parent
- o Biological parent
- o Foster parent or legal guardian
- o Grandparent or other family member
- o Step-parent
- o Relationship not listed:______
- o Prefer not to say

CC-BY 4.0 Crane, M. E., Atkins, M. A., Becker, S. J., Purtle, J., Olino, T. M., & Kendall, P. C. (2021)
doi.org/10.17605/OSF.IO/8X7B4

Does your child (initials:___) currently live with you?

- o Yes
- o No
- o Prefer not to say

What is the custody arrangement for your child?

- o Joint custody --- I share custody with another legal guardian
- o Sole custody --- I am the only legal guardian (even if remarried)
- o No formal custody arrangement
- o Custody arrangement not listed:______
- o Prefer not to say

What is the primary language spoken in your home?

- o English
- o Other:______
- o Prefer not to say

Number of children: _______

Number of children living in your home: _______

Highest education level completed:

- o Less than high school
- o High school graduate
- o College graduate (2-year)
- o College graduate (4-year)
- o Some college
- o Master's degree or equivalent
- o Doctoral degree or equivalent (MD, Ph.D., Psy.D., J.D.)
- o Prefer not to say

CC-BY 4.0 Crane, M. E., Atkins, M. A., Becker, S. J., Purtle, J., Olino, T. M., & Kendall, P. C. (2021)
doi.org/10.17605/OSF.IO/8X7B4

What religion are you? (check all that apply)

- ☐ Catholic
- ☐ Protestant
- ☐ Other Christian:____
- ☐ Jewish
- ☐ Buddhist
- ☐ Muslim
- ☐ Hindu
- ☐ Agnostic
- ☐ Atheist
- ☐ A religion not listed:_____
- ☐ Prefer not to say

How often do you usually attend religious services?

- o Never
- o Less than one a month
- o One to three times a month
- o About once a week
- o More than once a week
- o Prefer not to say

Total household income

- o Less than $15,000
- o $15,000-$24,999
- o $25,000-$34,999
- o $35,000-$49,999
- o $50,000-$74,999
- o $75,000-$99,999
- o $100,000-$149,999
- o $150,000-$199,999
- o $200,000 and over
- o Prefer not to say

CC-BY 4.0 Crane, M. E., Atkins, M. A., Becker, S. J., Purtle, J., Olino, T. M., & Kendall, P. C. (2021)
doi.org/10.17605/OSF.IO/8X7B4

Child demographics

**For the following questions, please enter your child's information (initials: _____) to the best of your knowledge.**

Age (in years): ___________

What sex was your child assigned at birth?

- ☐ Male
- ☐ Female
- ☐ A sex not listed:____
- ☐ Prefer not to say

How would your child describe their gender? (check all that apply)

- ☐ Agender
- ☐ Cisgender
- ☐ Female
- ☐ Genderqueer
- ☐ Male
- ☐ Non-binary
- ☐ Transgender
- ☐ Unsure
- ☐ A gender not listed:_____
- ☐ Prefer not to say

Sexual orientation (check all that apply):

- ☐ Asexual
- ☐ Bisexual
- ☐ Gay or Lesbian
- ☐ Queer
- ☐ Straight/heterosexual
- ☐ Unsure
- ☐ A sexual orientation not listed: ________
- ☐ Prefer not to say

CC-BY 4.0 Crane, M. E., Atkins, M. A., Becker, S. J., Purtle, J., Olino, T. M., & Kendall, P. C. (2021)
doi.org/10.17605/OSF.IO/8X7B4

Race (check all that apply):

- ☐ American Indian or Alaska Native
- ☐ Asian
- ☐ Black or African American
- ☐ Native American/Alaskan Native
- ☐ Native Hawaiian or Other Pacific Islander
- ☐ White
- ☐ A race not listed:_____
- ☐ Prefer not to say

Is your child of Hispanic, Latino/a/x, or of Spanish origin? (check all that apply)

- ☐ No, not of Hispanic, Latino/a/x, or Spanish origin
- ☐ Yes, Cuban
- ☐ Yes, Mexican, Mexican American, Chicano/a/x
- ☐ Yes, Puerto Rican
- ☐ Yes, another Hispanic, Latino/a/x or Spanish origin
- ☐ An ethnicity not listed:_____
- ☐ Prefer not to say

Where was your child born?

- o In the United States
- o In another country
- o Prefer not to say

Is your child covered by any of the following types of health insurance? (check all that apply)

- ☐ Health insurance obtained through an employer or union
- ☐ Health insurance purchased directly from an insurance company (e.g., healthcare.gov)
- ☐ Military health insurance
- ☐ Medicaid (e.g., PA Medical Assistance, ACCESS, NJ Family Care, Choose Health Delaware)
- ☐ CHIP
- ☐ Other:_____
- ☐ No
- ☐ Prefer not to say

CC-BY 4.0 Crane, M. E., Atkins, M. A., Becker, S. J., Purtle, J., Olino, T. M., & Kendall, P. C. (2021)
doi.org/10.17605/OSF.IO/8X7B4

**Knowledge Test**

Instructions: Please answer these questions to the best of your ability. If more than one question appears to be correct, please choose the best answer to the question. You are not expected to know all the answers. Please do not look up the answers. We want to know what you think off the top of your head.

1. Kids with anxiety are referred for help more often than kids with behavioral problems.
   a. True
   b. **False**

2. A child may refuse to go to school because something about school makes them anxious.
   **a. True**
   b. False

3. Trouble concentrating may be a sign of anxiety.
   **a. True**
   b. False

4. In the long-term, it is helpful for parents to protect their child from feelings of anxiety.
   a. True
   b. **False**

5. When some children feel nervous, they may ask the same question over and over again. To make the child less anxious in the long-term, the caregiver should answer the question each time it's asked.
   a. True
   **b. False**

6. Cognitive behavioral therapy is the most effective psychological treatment for anxiety.
   a. **True**
   b. False

7. The goal of therapy for anxiety is to get rid of a child's anxiety.
   a. True
   **b. False**

8. You should not put your child's name on many therapy waitlists at a time.
   a. True
   b. **False**

9. Therapists for youth anxiety all charge at least $100 per session.
   a. True
   **b. False**

CC-BY 4.0 Crane, M. E., Atkins, M. A., Becker, S. J., Purtle, J., Olino, T. M., & Kendall, P. C. (2021)
doi.org/10.17605/OSF.IO/8X7B4

10. Cognitive behavioral therapy takes at least a year to be effective.
    a. True
    b. **False**

11. Anxiety is a problem when
    a. It is experienced by a youth
    b. It is before or during a stressful experience
    **c. It causes a lot of distress or gets in the way of daily life**
    d. It is not fully experienced

12. Which of the following is most commonly seen in social anxiety disorder?
    a. Frequently asking caregivers if things will turn out okay
    **b. Trying to avoid social interactions**
    c. Worrying about heath
    d. Wanting to be close to a caregiver

13. A child may procrastinate homework because:
    a. They find homework to be boring
    b. They're worried about how well they will do on the assignment
    c. They want to complete the assignment quickly
    **d. A&B can both be true**

14. Anxious youth may ask questions about what will happen in the future. Which of the following is the most helpful way to think about uncertainty?
    a. When youth feel unsure, they should always seek answers to feel more certain
    **b. Children should learn to be okay with some amount of uncertainty about the future**
    c. Children should learn to toughen up and stop trying to feel more comfortable
    d. Wanting certainty about the future is a valuable skill to help them succeed

15. When a child feels anxious, as a caregiver, it is a good idea to do all of the following EXCEPT:
    **a. Change your behavior to reduce their anxiety**
    b. Accept that the child feels the emotion
    c. Reward them when they engage in brave behavior
    d. Model coping behaviors

16. Your child tells you they feel worried about an upcoming situation and asks not to go. What's the most helpful way for you to respond?
    a. Allow them not to go. There is no reason for them to feel distressed
    b. Have them face the situation head on and get it over with
    **c. Encourage them to approach the situation by breaking it down into manageable steps**
    d. Find an alternative situation that is less stressful

CC-BY 4.0 Crane, M. E., Atkins, M. A., Becker, S. J., Purtle, J., Olino, T. M., & Kendall, P. C. (2021)
doi.org/10.17605/OSF.IO/8X7B4

17. A child faced their fear of asking a friend to come over to hang out. Which of the following rewards could be used?
    a. 20 minutes extra screen time
    b. Getting to pick what's for dinner
    **c. Either A or B**
    d. Neither – children should not be rewarded for doing things they should be able to do

18. Which therapy strategy is most helpful for decreasing anxiety in the long-term?
    a. Deep breathing
    **b. Exposure (i.e., helping a child practice slowly facing their fears)**
    c. Coping thoughts
    d. Problem solving

19. How are caregivers usually involved with treatment?
    **a. Caregivers can help the youth practice skills at home between sessions**
    b. Caregivers should go to the same therapist as their child
    c. Caregivers should sit in on all of their youth's therapy sessions
    d. Caregivers should not get involved in the youth's "private and personal" treatment

20. How would you know if a therapist actually provides the most effective strategies used to treat youth anxiety?
    a. Ask if they provide cognitive behavioral therapy
    b. Ask if they conduct cognitive behavioral therapy with exposures
    c. Ask if they assign youth therapy homework between sessions
    **d. B & C are true**

CC-BY 4.0 Crane, M. E., Atkins, M. A., Becker, S. J., Purtle, J., Olino, T. M., & Kendall, P. C. (2021)
doi.org/10.17605/OSF.IO/8X7B4

**Scoring**

- Correct answer is bolded
- Items are scored such that 1 = correct; 0 = incorrect for a maximum of 20 points

CC-BY 4.0 Crane, M. E., Atkins, M. A., Becker, S. J., Purtle, J., Olino, T. M., & Kendall, P. C. (2021)
doi.org/10.17605/OSF.IO/8X7B4

**Project CHAT Key Opinion Leader Training Checklist**

*Check off the following items if they occurred:*

1. Sent key opinion leaders (KOLs) ppt ahead of time to review
   - o Yes
   - o No

**Group Training**

- ☐ Discuss what made them want to be involved with the presentation
- ☐ Discuss their experiences with anxiety
- ☐ Discuss anxiety in their community
- ☐ Review cycle of avoidance
- ☐ Review exposure therapy
- ☐ Review presentation
- ☐ Get feedback on presentation
- ☐ Discuss what strategies they could endorse

Training length (minutes): _______

**Presentation Modification**

- ☐ Presentation modified based on KOL feedback

**Phone Call**

- ☐ Review/approved modifications made
- ☐ Answer KOL questions about the content
- ☐ Determine which sections the KOL is comfortable presenting
- ☐ Determine which strategies the KOL is willing to endorse
- ☐ Give the KOL an opportunity to practice to ensure comprehension

Phone call length (minutes): ____________

Notes about KOL Training:_____________

CC-BY 4.0 Crane, M. E., Atkins, M. A., Becker, S. J., Purtle, J., Olino, T. M., & Kendall, P. C. (2021)
doi.org/10.17605/OSF.IO/8X7B4

## Project CHAT Presentation Content Checklist

Who is filling out this checklist?:_______

**Presentation Length**
Start time: ______________________
End Time: _______________________
Presentation time (minutes): __________________
Q&A start time:______________
Q&A length (minutes):__________

**Presenter Speaking Time**
Presenter 1 (Margaret) speaking time (minutes): ________
Presenter 2 speaking time (minutes): ___________

Which sections of the presentation did Presenter 2 give?

- ☐ Recognizing anxiety
- ☐ Strategies for caregivers
- ☐ Seeking additional support

Which sections of the presentation did Presenter 2 provide comments during?

- ☐ Recognizing anxiety
- ☐ Strategies for caregivers
- ☐ Seeking additional support
- ☐ Q&A

**Anxiety Overview**

- ☐ Overview of anxiety
- ☐ Separation anxiety
- ☐ Social anxiety
- ☐ Generalized anxiety
- ☐ COVID anxiety
- ☐ Physical symptoms of anxiety
- ☐ Behavioral problems
- ☐ School refusal
- ☐ Avoidance overview

CC-BY 4.0 Crane, M. E., Atkins, M. A., Becker, S. J., Purtle, J., Olino, T. M., & Kendall, P. C. (2021)
doi.org/10.17605/OSF.IO/8X7B4

**Strategies for parents**

- ☐ Label and validate a child's emotions
- ☐ Break down anxious situations into small steps
- ☐ Avoid unnecessary accommodations
- ☐ Responding to reassurance seeking
- ☐ Reward brave behavior
- ☐ Stay calm

**Seeking additional support**

- ☐ When is treatment needed?
- ☐ CBT overview
- ☐ Medication treatment
- ☐ How to find a therapist
- ☐ Questions to ask a therapist
- ☐ Ask for exposure therapy
- ☐ Process of starting therapy

**Did any of the following disclosures occur?**

| Disclosure | Margaret | Presenter 2 | Example about another family | Participant self-disclosure (e.g., during Q&A) |
|---|---|---|---|---|
| Receiving therapy for self | | | | |
| Receiving CBT for self | | | | |
| Having anxiety | | | | |
| Child receiving therapy | | | | |
| Child receiving CBT | | | | |
| Child having anxiety | | | | |
| None | | | | |

**Other**

Were the handouts send in the chat

- ☐ Yes
- ☐ No

Notes:______________

CC-BY 4.0 Crane, M. E., Atkins, M. A., Becker, S. J., Purtle, J., Olino, T. M., & Kendall, P. C. (2021)
doi.org/10.17605/OSF.IO/8X7B4

## Project CHAT Qualitative Interview Guide

Thank you for agreeing to participate. As part of this study, you attended a presentation on youth anxiety. Other words for anxiety can be fears, worries, feeling nervous, being afraid, and so on.

This presentation talked about to identify anxiety, strategies for caregivers, and how to find a therapist. Today, I will ask about your thoughts of the presentation and your experiences seeking therapy for your child. I have prepared some questions that will take approximately 30 minutes to answer. You can spend as much or as little time as you like answering each question. It's important for you to know that there are no right or wrong answers. We are interested only in *your* opinions and perceptions. After the interview, you will be sent $20 for your participation. Do you have any questions?

### Role of Presenters

For the first few questions, I'm going to ask you about the people who gave the presentation. *Researcher only-condition:* As a reminder, the presenters from Temple were named Margaret and [presenter 2].

*Key Opinion Leader (KOL) condition:* As a reminder, the presenter from Temple was named Margaret. The presenter from your school was named [name].

1. (Perception of presenters)

    *For researcher-only*
    - What was your perception of the presenters?
    - In what ways were the presenters a good source of information?
    - In what ways weren't the presenters a good source of information?

    *For KOL*
    - What was your perception of Margaret (the presenter from Temple)
    - In what ways was Margaret a good source of information?
    - In what ways wasn't Margaret a good source of information?
    - What was your perception of [KOL name] (the parent from your school)?
    - In what ways was [KOL name] a good source of information?
    - In what ways wasn't [KOL name] a good source of information?

2. (Presenter role in seeking treatment)

    *For the researcher-only condition:*
    - What role the presenters play in your decision to seek treatment?
    - How did hearing from researchers change your perception of seeking therapy?
        - <u>Optional</u>: How did the presenters change how you felt about the way others might view you if you seek therapy for your child?

    *For the KOL condition:*
    - What role did Margaret (the presenter from Temple) play in your decision to seek treatment?
    - How did hearing from a researcher change your perception of seeking therapy?

CC-BY 4.0 Crane, M. E., Atkins, M. A., Becker, S. J., Purtle, J., Olino, T. M., & Kendall, P. C. (2021)
doi.org/10.17605/OSF.IO/8X7B4

    - Optional: How did Margaret change how you felt about the way others might view you if you seek therapy for your child?
- What role did [KOL name] (the parent from your school) play in your decision to seek treatment?
- How did hearing from another caregiver change your perception of seeking therapy?
    - Optional: How did [KOL name] change how you felt about the way others might view you if you seek therapy for your child?

**Factors for Choosing a Therapist**

3. What factors were most important when considering whether or not to seek therapy for your child?

   *If has sought therapy*

   - What factors did you consider when choosing the therapist?

**Presentation**

4. What strategies from the presentation have you used to help your child's anxiety?

5. Was presentation content relevant for you? *If yes*, how was it relevant? *If no*, what didn't feel relevant?

6. Optional: What information did the presentation give you that helped/would help you seek therapy for your child?

**Perception of Exposure Therapy**

7. One strategy therapists use is called exposure therapy, where the therapist helps a child slowly face their fears. What is your perception on this kind of therapy?
   - How did the presentation shift your perception of exposure therapy?
   - How could we describe exposure therapy to encourage parents to seek such treatment?
       - Optional prompt: What would be a better term for this type of therapy?
   - Optional: How would you find a therapist who uses exposure therapy?

**Other**

8. Optional: Have you had any previous experiences with therapy? If so, can you please explain how this may affect what therapy you would seek for your child?

9. If you could wave a magic wand and change the mental health system to make it easier for families to seek therapy for their children, what would you change?

10. Do you have any additional feedback for us?

Additional prompt throughout: If a participant talks about seeking therapy, ask if they sought a specific type of therapy.

CC-BY 4.0 Crane, M. E., Atkins, M. A., Becker, S. J., Purtle, J., Olino, T. M., & Kendall, P. C. (2021)
doi.org/10.17605/OSF.IO/8X7B4

**INTERVIEWER COMMENTS**

1. Respondent's level of interest and involvement in answering questions.

| | | | | |
|---|---|---|---|---|
| 1.<br>Very low | 2.<br>Low | 3.<br>Neutral | 4.<br>High | 5.<br>Very high |

2. Please estimate the respondent's understanding of the interview.

| | | | | |
|---|---|---|---|---|
| 1.<br>Limited | 2.<br>Partial | 3.<br>Average | 4.<br>Majority | 5.<br>Complete |

3. Please rate your impression of the knowledge of the respondent in the topic being addressed in this module.

| | | | | |
|---|---|---|---|---|
| 1.<br>Highly Questionable | 2.<br>Somewhat Questionable | 3.<br>Neither | 4.<br>Somewhat Knowledgeable | 5.<br>Highly Knowledgeable |

4. Describe any discrepancies, gaps, or other problems with the interview.

5. Describe any circumstances that occurred while the interview was in progress that may have affected the quality of the interview (i.e., interruptions)?

6. Describe any affective and/or non-verbal responses displayed by the participant:

CC-BY 4.0 Crane, M. E., Atkins, M. A., Becker, S. J., Purtle, J., Olino, T. M., & Kendall, P. C. (2021)
doi.org/10.17605/OSF.IO/8X7B4
